# Ultrasonic pulse-echo dataset from numerical modelling for oil and gas well integrity investigations

**DOI:** 10.1038/s41597-025-04851-x

**Published:** 2025-04-01

**Authors:** Anja Diez, Erlend Magnus Viggen, Tonni Franke Johansen

**Affiliations:** 1 SINTEF AS, Sustainable Communication Systems, Acoustics, Trondheim, Norway; 2 Norwegian University of Science and Technology (NTNU), Department of Circulation and Medical Imaging, Trondheim, Norway

**Keywords:** Energy science and technology, Imaging techniques, Electrical and electronic engineering

## Abstract

The ultrasonic pulse-echo (PE) measurement is a crucial measurement technique to determine the integrity of oil and gas wells. Oil companies use various analysis techniques and corrections to derive the pipe thickness and impedance of the material behind the pipe from PE measurements that are carried out inside the pipe. While some field measurements are publicly available, they have no corresponding ground truth. We therefore simulated a dataset of PE measurements with ground truth. The dataset was generated using axisymmetric models and 3D models in COMSOL Multiphysics. The base geometry was based on common parameters from the field: oil-based mud on the inside of a 9.625 in pipe and cement on the outside of the pipe. From this base geometry, variations in the model parameters were introduced, for example, plate/pipe wall thickness, different materials on both sides of the wall, different pipe diameter, different annulus thicknesses, eccentering. The generated dataset allows detailed investigations of existing PE analysis algorithms, comparison of those and development of new PE analysis techniques.

## Background & Summary

Pulse-echo (PE) ultrasonic measurements are used in oil industry to determine the material behind the pipe and its condition, determine the pipe thickness, and detect possible fluid filled annuli. Different analysis methods have been proposed by different oil companies^1–4^ based on comparisons of measurement data with results from a 1D plane wave model and including a range of corrections to derive the impedance of the material behind the pipe. However, these methods differ both in their analysis method and in which part of the received signal these methods use to derive pipe thickness and outer-material impedance. It is therefore of interest to have the possibility to quantify the results of the different algorithms, possibly combine them, and improve them or develop new analysis algorithms. This requires data with ground truth information on thickness and impedance. Further, to quantify the effect of different variations in geometry and/or material parameters it is beneficial to have a dataset with variations in only one parameter at a time. Both are normally not available in measured data from the field. Therefore, we generated the here presented simulated dataset^5^ using COMSOL Multiphysics based on commonly used pipe geometries and material parameters.

This simulated dataset^5^ was created to allow to: (1) study the performance of these industry algorithms, (2) investigate how reliably they estimate the impedance of the material behind the pipe in the presence of geometry deviations such as pipe thickness and transducer-pipe distance variations, (3) investigate the different corrections required by today’s algorithms, and (4) allow for the development of improved PE analysis techniques for well applications. This dataset^5^ provides simulated measurements with ground truth, as the estimated parameters can be compared to the known parameters from the model setup.

In well integrity investigations, the ultrasonic PE measurement is a common technique to estimate the casing thickness, the material behind the well pipe, and the presence of microannuli. An ultrasonic pulse is sent from the inside of the pipe onto the pipe wall and the reflected signal recorded at the same transducer is analyzed. Such ultrasonic PE measurements are not direct measurements of pipe thickness and material parameters but estimations derived through frequency analysis and comparison to 1D modelling.

This dataset of PE data^5^, generated using numerical modelling in COMSOL Multiphysics, contains a large range of variation in model parameters to study the effect of these variations on the ultrasound signal and the results derived from it. Most of the modelling was done in axisymmetric 3D geometries including model parameter variations such as pipe thickness, pipe/plate geometry, material parameters inside and outside the pipe, and pipe diameter. This data is referred to as 2.5D data, to distinguish it from data from simulations using either pure 2D or full 3D models. This was supplemented with full 3D modelling to study varying degrees of eccentering^6^. The dataset is published on Mendeley Data^5^ and contains COMSOL model files to reproduce the data, text files with the raw data output from COMSOL, and JSON files with the derived waveform and all relevant metadata to set up the corresponding simulation.

## Methods

**Fig. 1:**
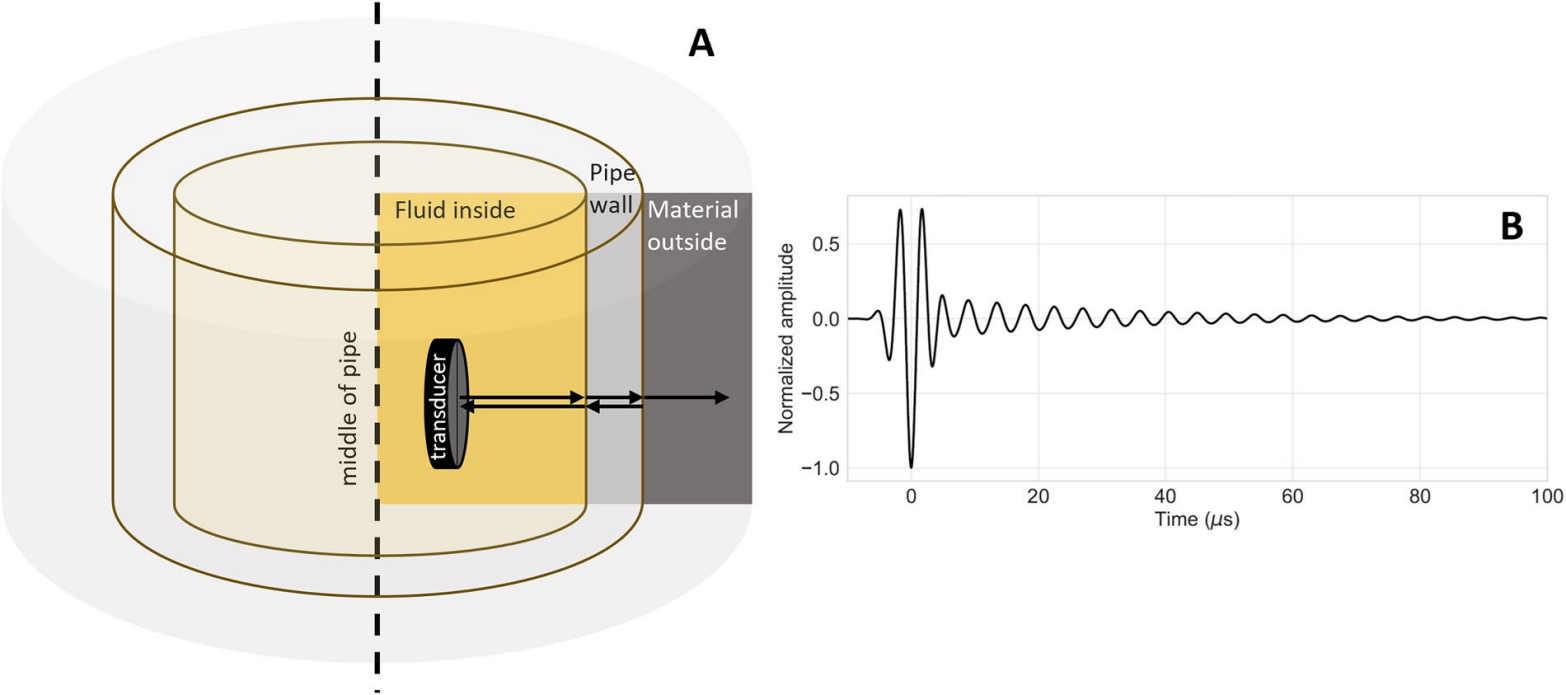
(**A**) Concept of PE measurements with a transducer insider the pipe. (**B**) Example of a signal as recorded by the transducer inside the pipe.

In PE measurements a circular ultrasound transducer is placed inside the well pipe perpendicular to the pipe wall. The transducer sends an ultrasound pulse under normal incidence towards the pipe. Most of the pulse energy is reflected from the pipe’s inner surface, while part of the pulse energy is transmitted into the pipe wall. Inside the pipe wall, the pulse reverberates between the pipe wall’s inner and outer surfaces. At each reflection, some energy is transmitted to the outside and some reflected to the inside of the pipe (Fig. 1A). The transducer inside the pipe records the combined signal from the initial strong reflection and the reverberations inside the pipe wall (Fig. 1B). The amplitude decay of the reverberating part of the signal depends on the amount of energy transmitted to the outside of the pipe wall with each reflection within the pipe wall, and hence, contains information on the material outside the pipe. This decay can, therefore, be analyzed to determine the impedance of the material behind the pipe. For a detailed description and mathematical derivation of the relevant equations, see, e.g., the books by Kinsler *et al*.^7^ and Kuttruff^8^.

To generate such a PE dataset^5^ where the geometrical and material parameters are known (ground truth) we used finite-element numerical modelling based on the software COMSOL Multiphysics making use of the acoustic and structural mechanics modules of this software package. The COMSOL software was used to generate this dataset of PE measurements for models reflecting common pipe dimensions and transducer settings.

### Model Setup

**Fig. 2:**
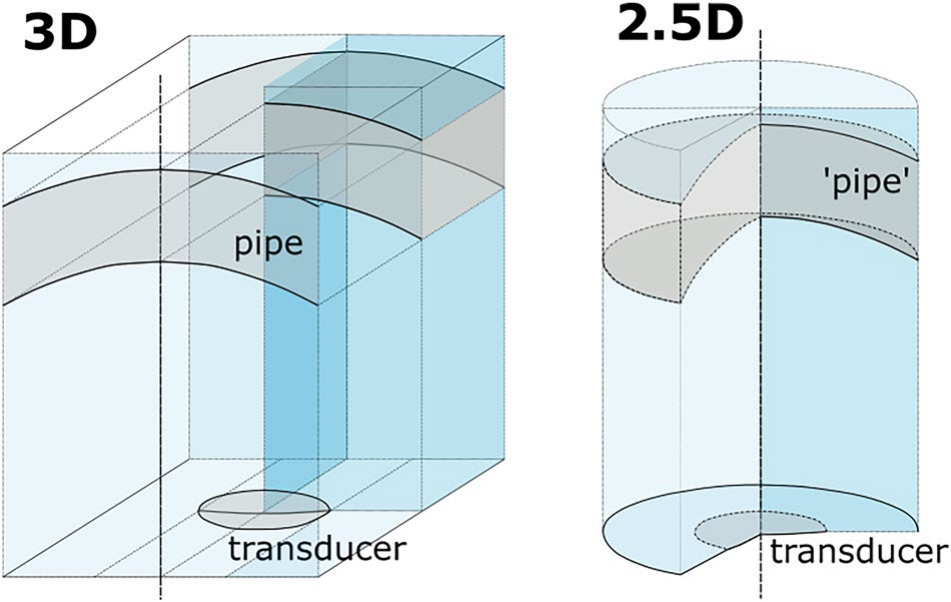
Sketch of 3D and axisymmetric 2.5D models and their symmetries. The darker colored parts indicate the area modelled in COMSOL when the symmetries were utilized.

**Fig. 3:**
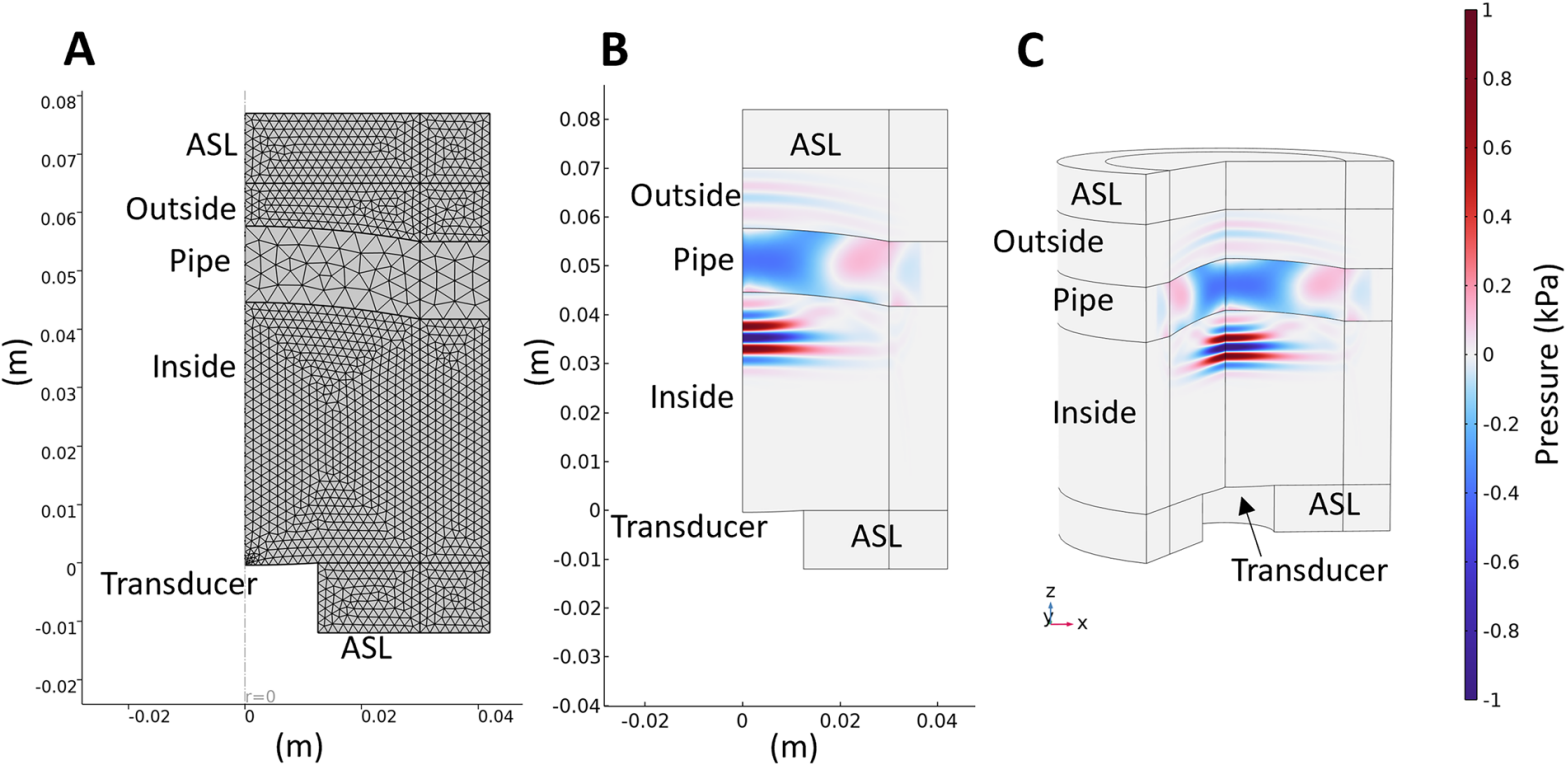
Example of axisymmetric 2.5D COMSOL model with mud inside and outside of the pipe. bf(A) shows the grid, bf(B) the 2D modelling plane, and bf(C) the corresponding extension in 3D of the axisymmetric model at the modelling timestep of 50 *μ*. Absorbing layers (ASL) surround the model domain.

The base geometry was set up as axisymmetric 3D models, referred to as 2.5D models in the following. In these models, we define the geometry of the model in a 2D cross section of the 3D geometry, perpendicular to the length axis of the pipe (Fig. 2), assuming azimuthal symmetry to reduce the computational requirements of the model. The transducer is defined as a parametric curve taking the diameter and the focal length into account. The pipe is defined via circle segments. The modelling domain is surrounded by an absorbing layer (Fig. 3A) to avoid reflection from the model boundary. The transducer surface is defined as an outer boundary on which the boundary load is defined as a Gaussian pulse with a cosine apodization over the transducer radius to avoid boundary effects at the outer edge of the transducer.

Boundaries between solid and fluid domains were not joined. For the modelling we primarily used COMSOL’s time-explicit formulation coupling the acoustic and structural-dynamics domains through their interfaces without any requirement of mesh continuity (Fig. 3A). The models were calculated for a length of 140 *μ*, with data being stored for every 0.5 *μ*. Figure 3B and C show the same result after 50 *μ* of wave propagation, with B showing the modelled 2D cross-section and C the axisymmetric extension into full 3D.

Modelling in 2.5D introduces an error in the pipe geometry which is modelled as a spherical shell with the same radius, while the transducer geometry is modelled correctly. Diez *et al*.^9^ showed in a comparison of 2.5D and 3D results that 2.5D can deliver reliable results down to a pipe diameter of 9 in. For the models with a plate instead of a pipe the geometry is modelled correctly with a 2.5D model. A comparison of different geometries and domains showed that 2.5D plate models can be a valuable approximation for 3D pipe models^9^, and plate models were therefore also included in this dataset. Reducing the modelling domain from 3D to 2.5D reduces each model’s computation time from multiple hours to about 10 min and is therefore preferable when building such a dataset. We therefore build the majority of this dataset using 2.5D models and supplement this with some 3D model results, in particular when simulating eccentering.

When studying eccentering, the transducer and pipe location are shifted relative to each other, hence there is only one symmetry plane in the 3D geometry. Eccentering is the main variation that needs to be modelled in 3D and cannot be approximated by a 2.5D model.

When introducing an annulus, i.e., a gap between the pipe and the surrounding formation (cement or shale) we used the time domain formulation. When introducing a fluid filled microannulus, the time-explicit formulation requires a lot of additional computational power to calculate the coupling of the acoustic and solid domain. Here, we used the time domain formulation, which requires mesh continuity, as it is a lot more efficient for this case^9^.

The exported raw data comprises the pressure over time at multiple points across the transducer surface. For the 2.5D models, these points lie along the circular arc defined by the transducer focal length and diameter, and for the 3D models, the points are distributed over the transducer surface. To derive an overall waveform for the entire 3D transducer surface, the pressures at the different points are averaged so that the pressure at a point is weighted proportionally to the area of the surface patch corresponding to that point. In the case of the 2.5D model, each point is weighted with the area of its corresponding circular ring segment to derive one waveform trace (Fig. 1A) from each COMSOL model run.

### Model Variations

A range of different materials that can be expected in a borehole are used in the COMSOL models: steel for the pipe, oil-based mud (obm) and water-based mud (wbm), pressurized methane as gas, shale as formation, and cement. Their material parameters are given in Table 1. The methane parameters were intended to be roughly representative but not exact (gas parameters also vary strongly with pressure and temperature) and were based on standard-condition parameters extrapolated to 100 bar and 60°C using an ideal-gas approximation. The values for oil-based mud and water-based mud are taken from Molz *et al*.^10^. Here we choose values from opposite ends of the spectrum to see more variations in our model results. Material parameters for steel, cement, and shale are taken from Diez *et al*.^9^.

**Table 1:** Material properties used in the COMSOL model.

Material	P-wave speed	S-wave speed	Density	Poisson’s ratio	Young’s modulus	Bulk modulus	Impedance
	m/s	m/s	kg/m^3^		GPa	GPa	MRayl
Methane	47	—	58	0.5	0	0.013	0.028
Oil based mud	1301	—	1250	0.5	0	2.12	1.63
Water based mud	1360	—	2160	0.5	0	4	2.94
Shale	2100	1000	2110	0.3657	5.41	9.31	4.43
Cement	3350	1920	1980	0.2554	18.33	12.49	6.63
Steel	5847	3160	7980	0.2937	206	158	46.66

The modelled variations were chosen as a deviation from a base model, where we defined a base model for a plate wall and one base model with a pipe wall. The following dimensions were used in these base models, modelled as 2.5D time-explicit model:

- wall: steel plate or pipe with 9.625 in diameter and 13 mm thickness
- transducer: focal length 20 cm, diameter 2.5 mm,
- acoustic pulse: Gaussian pulse, bandwidth 0.7, center frequency 250 kHz,
- inside wall: oil-based mud, transducer-pipe distance of 45 mm,
- outside wall: cement.

Variations in the geometry were used to derive data for a large range of different conditions that can be expected in an oil and gas well. The general variations and their combination are listed in Table 2. This table gives an overview of the modelled combined variations, which are indicated with checkmarks. E.g., column no. 1 and row no. 2 show that thickness variations were modelled for both plate and pipe geometries. Dashes are placed along the diagonal of this symmetric table to indicate cells that do not correspond to combinations of variations, while the empty cells mark combinations of variations that were not modelled.

**Table 2:** Overview of simulated combinations of model variations.

		Variations	1	2	3	4	5	6	7	8
**1**	Geometry interface	plate, pipe	—	*✓*	*✓*	*✓*		*✓*	*✓*	*✓*
**2**	Thickness	7.4:0.2:16.6 mm	*✓*	—	*✓*	*✓*	*✓*	*✓*		
**3**	Material behind	methane, obm, wbm, shale, cement	*✓*	*✓*	—	*✓*	*✓*	*✓*	*✓*	*✓*
**4**	Material inside	obm, wbm	*✓*	*✓*	*✓*	—	*✓*	*✓*		
**5**	Diameter pipe (2.5D)	7, 9.625, 10.75, 13.375, 20 in		*✓*	*✓*	*✓*	—	*✓*	*✓*	*✓*
**6**	Annular gap	10:10:90 *μ*m, 100:100:1000 *μ*m	*✓*	*✓*	*✓*	*✓*	*✓*	—		
**7**	Distance	35:5:55 mm	*✓*		*✓*		*✓*		—	*✓*
**8**	Eccentering 3D	1^°^, 3°, 5^°^	*✓*		*✓*		*✓*		*✓*	—

The thick lines separate the different model types: We used 2.5D time-explicit models for variations 1–5, 2.5D time-domain models for variations 6, and 3D time-explicit model for variations 7–8. The variations column refers to geometry and material changes in the model as described in the Model Variations section. “*✓*” indicates combinations of variations that were modelled, “—” is placed along the diagonal to indicate cells that do not correspond to variation combinations, and empty cells indicates combinations that were not modelled.

## Data Records

The data has been published via Mendeley Data (10.17632/3bs65nzpv2.1). The dataset^5^ contains:

- COMSOL model setup as .mph file used to generate the dataset
- Time series data as .txt files containing the raw data
- Processed time series data (waveform trace) with accompanying metadata as .json files

The data, which were generated using COMSOL Multiphysics, are saved as text files and in the text-based and human-readable JavaScript Object Notation (JSON) file format. From each simulated pulse-echo COMSOL model, results are derived and saved in: (i) a .txt file exported from COMSOL containing the raw data, that is the acoustic pressure over time at points across the transducer surface, and (ii) a .json file containing the metadata giving all the relevant parameters describing the model domain (Table 3) and the waveform. This waveform is calculated from the raw pressure data in the .txt file through a weighted averaging over the transducer area as described above. These files are named with a numerical identifier. JSON files (1.json) and raw data files (1.txt) corresponding to the same model use the same identifier.

**Table 3:** Description of metadata included in the JSON files.

Category	Parameter name	Description
general	domain	2.5D (axisymmetric model), or 3D
	interface	time-domain or time-explicit
	geometry	plate or pipe
	symmetries	axisymmetric, 2 symmetry axes, or 1 symmetry axis
	height	domain height in m
	width	domain width in m
	depth	domain depth in m (only for 3D)
grid	definition	definition to calculate max grid size
	fg	grid reference frequency in Hz
boundary	type	perfectly matched layer or absorbing layer
	element_no	number of elements in case of perfectly matched layer
	thickness	thickness of the boundary domain in m
transducer	focal_length	transducer focal length in m
	diameter	transducer diameter in m
	apodization	transducer apodization type for transmission
pulse	type	pulse type, e.g., Gaussian pulse
	bw	bandwidth of pulse
	f0	center frequency of pulse in Hz
	definition	equation of pulse definition
	tp0	time of the envelope maximum of the transmitted pulse in s
inside	material	Material inside the wall (pipe or plate)
	rho	density of material inside the wall in kg/m3
	vp	compressional wave speed of material inside the wall in m/s
	vs	shear wave speed of material inside the wall in m/s
	transducer_ pipe_distance	distance between center of transducer and wall interface in m
interface	material	material of wall (pipe or plate)
	rho	density of wall material in kg/m3
	vp	compressional wave speed of wall material in m/s
	vs	shear wave speed of wall material in m/s
	inner_diameter	inner diameter of pipe in m (null if plate)
	outer_diameter	outer diameter of pipe in m (null if plate)
	thickness	thickness of wall in m
outside	material	material outside the wall (pipe or plate)
	rho	density of material outside the wall in kg/m3
	vp	compressional wave speed of material outside in m/s
	vs	shear wave speed of material outside the wall in m/s
gap	material	material inside the gap (annulus)
	rho	density of material of the gap in kg/m3
	vp	compressional wave speed of material of the gap in m/s
	vs	shear wave speed of material of the gap in m/s
	thickness	thickness of the gap in m
eccentering	shift	shift of the tool center in m
	transducer_angle	angle of transducer in relation to axis of tool shift in degrees
	incident_angle	angle of incidence on interface surface in degrees
	transduce_pipe_distance	actual distance between interface and transducer in case of eccentering in m
waveform	t0	start time of time vector for data in s
	dt	time step of the time vector for the data in s
	sample_no	length of data vector
	values	data vector, normalized amplitudes (to maximum amplitude)

The first parts of the JSON file contain the metadata of the model, to allow full reproduction of the dataset^5^. Table 3 gives an overview over the different categories. Each category contains parameters defining a specific part of the model. Explanations of these parameters and the used SI units are given in Table 3. The category ‘waveform’ contains the vector ‘values’ that gives the normalized signal data for the trace calculated from the model described in the metadata.

The entire list of model parameter variations used in the COMSOL models to generate this dataset is given in the table file https://data.mendeley.com/datasets/3bs65nzpv2/1/files/033e628a-da39-4909-9e72-c306484c7264 ‘Metadata_overview.csv’ included in the dataset^5^.

## Technical Validation

Extensive investigation of the consistency of these model results were carried out by Diez *et al*.^9^ before building the dataset^5^. Correctly capturing a geometry in a model with a circular transducer inside an elongated pipe (Fig. 2) requires a 3D model. Models without eccentering can use two symmetry planes, so that only a quarter of the pipe/transducer geometry needs to be modelled, as indicated by the darker colored quarter in Fig. 2. However, 3D models are computationally heavy due to the distance between transducer and pipe (around 45 mm) in combination with the signal’s frequency (250 kHz) and the, thus, required grid size. Therefore, 3D models were not suitable to build a dataset of this size, even when making use of the symmetries. In a comparison of 1D, 2D, 2.5D (axisymmetric) and 3D model results we found that 2.5D (axisymmetric) models (Fig. 2) can deliver reliable results down to a pipe diameter of 9 in^9^. In the case of a 2.5D model, the circular transducer is modelled correctly and if a plate is used as wall the plate geometry is modelled correctly too. However, the pipe is modelled as a spherical shell and not a pipe in a 2.5D model. Nevertheless, results of the 2.5D models agree well with those of the 3D models^9^.

The 2.5D and 3D model waveforms were also compared to the 1D model waveforms^9^ that are used in prominent industry algorithms as comparison data in the inversion step^1–4^. However, it is known that the 1D models introduce errors due to the reduction from 3D to 1D and impedance and thickness values derived with these algorithms require corrections^1^. The discrepancies between 2.5D and 3D with the 1D model observed by Diez *et al*.^9^ are therefore expected, while general trends of the waveforms and of the ringdown agree well.

Direct comparison to real downhole data is only qualitatively possible as the real data has no ground truth and as the exact waveform will change depending on the used pulse form and frequency. However, generally good agreement can be observed when comparing the COMSOL model data with data from down-scaled lab experiments^11^.

## Usage Notes

The JSON data files of the dataset^5^ have been generated for easy access and usability and containing all the metadata. It can be opened with any text editor, containing the information described in Table 3. The .txt files contain the COMSOL output related to the corresponding .json file those allowing full flexibility in averaging the data used to generate the PE measurement trace. The open source Python toolbox Pyintegrity (https://github.com/erlendviggen/pyintegrity) can read the .json data files into Python and implements some of the most common analysis algorithms^12^. This allows for fast and convenient import of the data to Python and initial analysis if required.

## Data Availability

The data has been published on Mendeley Data (10.17632/3bs65nzpv2.1). The dataset repository also contains the COMSOL model setup as .mph files. These describe the entire model setup used to generate the dataset and allow reproducing the dataset or extending it. The metadata in the .json files contains the parameters used to set up and vary these models.
